# Host norepinephrine biases codon-dependent virulence translation in EHEC

**DOI:** 10.1128/msystems.00384-26

**Published:** 2026-05-14

**Authors:** Zeynep Baharoglu

**Affiliations:** 1 Epitranscriptomic and translational responses to antibacterial stress Team, Université Paris Cité, CNRS, Institut de Biologie Physico-Chimique, Expression Génétique Microbienne, Institut Pasteur 27058, Paris, France; Chinese Academy of Sciences, Shanghai, China

**Keywords:** epitranscriptome, tRNA modification, virulence regulation, codon usage

## Abstract

Bacterial pathogens sense host-derived signals to control virulence, yet how these cues shape translation remains unclear. McShane et al. show that the host hormone norepinephrine rewires the tRNA epitranscriptome of enterohemorrhagic *Escherichia coli* (EHEC) O157:H7, altering wobble-position modifications and shifting decoding capacity toward A/U-ending codons (A. E. C. McShane, C.-K. Chan, R. Chen, M. S. DeMott, et al., mSystems 11:e01418-24, 2026, https://doi.org/10.1128/msystems.01418-25). This bias aligns with the codon architecture of the locus of enterocyte effacement (LEE), a major virulence locus, enabling selective translation of virulence genes. Multi-omic analyses link these changes in tRNA chemistry to proteome remodeling without major shifts in tRNA abundance. By coupling host hormone sensing to codon-biased translation, this work establishes the tRNA epitranscriptome as a dynamic regulator of bacterial virulence.

## COMMENTARY

Understanding how bacterial pathogens interpret host-derived signals remains a central challenge in microbial pathogenesis. In enteric environments, pathogens, such as enterohemorrhagic *Escherichia coli* (EHEC), must integrate diverse cues, including nutrients, microbiota-derived metabolites, immune effectors, and host neuroendocrine signals, to precisely regulate virulence programs. While the transcriptional networks governing these responses have been extensively characterized, particularly for catecholamine sensing (1, 2), how these cues influence translation remains far less clear.

This gap is increasingly relevant as translation is no longer viewed as a passive endpoint of gene expression, and mRNA and protein levels do not always correlate (3). Transfer RNAs (tRNAs), short non-coding RNAs central to decoding the genetic code, are extensively modified, particularly at the anticodon loop where wobble base pairing occurs (4). These modifications, ranging from simple methylations to complex structures, such as queuosine, directly influence decoding efficiency, fidelity, and codon preference (5). Once considered static, tRNA modifications are now recognized as dynamic and responsive to environmental conditions, including stress, nutrient availability, oxygen, and growth state (6). In this context, the tRNA epitranscriptome emerges as a regulatory layer capable of rapidly reshaping proteome composition.

A key conceptual advance in the field is modification tunable transcripts (MoTTs), genes whose codon usage renders their translation particularly sensitive to changes in tRNA modification status (7). These transcripts, often enriched in specific or “rare” codons, can be selectively translated under stress conditions when the corresponding tRNA modifications are altered, allowing prioritization of stress-response or virulence-associated proteins. Although this paradigm has been supported in contexts, such as oxidative stress and antibiotic exposure (reviewed in references 8, 9), direct links to defined host signals have remained limited.

McShane et al. address this gap by linking a well-characterized host cue, norepinephrine, to tRNA modification dynamics and codon-biased translation in EHEC (10). Norepinephrine, which is abundant in the gut, stimulates chemotaxis, motility, and type III secretion system (T3SS) through signaling pathways that converge on transcriptional regulation, primarily involving two-component systems (TCS) and quorum-sensing circuits (11). Here, norepinephrine exposure drives coordinated remodeling of the tRNA epitranscriptome, with measurable consequences for proteome composition.

A central insight of the work is the functional reinterpretation of codon usage in virulence loci. The locus of enterocyte effacement (LEE), long recognized for its atypical GC content and codon bias (12), is shown here to be strongly enriched in A/U-ending codons. Under norepinephrine exposure, multiple wobble-position tRNA modifications, including 5-hydroxyuridine derivatives (m)cmo^5^U, queuosine, and inosine, are altered in ways that shift decoding capacity away from G/C-ending codons. This generates a translational environment that favors the codon architecture of LEE genes, consistent with their behavior as modification-tunable transcripts. More broadly, these findings define a pathway in which host signaling reshapes tRNA modification states, alters decoding capacity, and biases translation of codon-structured virulence programs.

The strength of this model lies in its multi-omic support. By integrating codon usage analysis, LC-MS-based quantification of tRNA modifications, absolute tRNA abundance measurements, and quantitative proteomics, the study establishes a direct link between RNA chemistry and protein output. Notably, these effects are driven by changes in tRNA modification status rather than large shifts in tRNA abundance, indicating that EHEC adapts translation through rapid tuning of decoding properties rather than restructuring of the tRNA pool. Consistent with this, reductions in cmo⁵U- and mcmo⁵U-related modifications, along with decreases in queuosine and inosine, are predicted to impair decoding of G/C-ending codons; accordingly, proteins downregulated under norepinephrine exposure are enriched in such codons, whereas upregulated proteins tend to avoid them.

These findings align with a broader body of work linking tRNA modifications to bacterial fitness and virulence. Pathways, such as MnmA-, GidA/MnmE-mediated wobble uridine modification, as well as Tgt-dependent queuosine incorporation, influence virulence gene expression, stress resistance, and host colonization across diverse pathogens (13–22). While disruption of these pathways often impairs motility, adhesion, or intracellular survival, the mechanistic basis has frequently remained unclear. This study provides a framework for how host-induced changes in tRNA modification states can directly bias translation of codon-structured virulence programs.

The study further integrates tRNA modification dynamics with cellular metabolism, by linking changes in 5-hydroxyuridine-derived modifications and carboxy-SAM (*S*-adenosylmethionine) levels to prephenate metabolism. This is consistent with the observed decrease in key shikimate pathway enzymes (e.g., PheA, AroH, and AroF) and places the tRNA epitranscriptome within central metabolic pathways, including aromatic amino acid biosynthesis. This supports the emerging view that RNA modification enzymes are metabolically embedded and responsive to shifts in cellular physiology, suggesting that the translational response to norepinephrine reflects both signal transduction and metabolic reprogramming.

Despite these advances, host-pathogen interactions viewed through the lens of tRNA modification remain underexplored, in part due to historical technical challenges in detecting and quantifying RNA modifications (23). As these approaches continue to improve, similar regulatory strategies are likely to emerge across diverse pathogens and environmental contexts.

Taken together, McShane et al. demonstrate how host-derived signals can shape bacterial gene expression beyond transcription. By linking norepinephrine sensing to tRNA modification dynamics and codon-biased translation, this study expands the regulatory framework of EHEC pathogenesis and positions the translation machinery as an active mediator of host-pathogen interactions. Rather than simply activating virulence genes transcription, EHEC remodels its decoding landscape in a manner that favors their translation, revealing epitranscriptomic control as a central and dynamic layer of adaptive virulence regulation.

Several questions remain open. First, the mechanisms by which norepinephrine modulates tRNA pools remain to be elucidated, potentially involving metabolic rewiring and altered availability of biosynthetic precursors. Second, while the study provides important insights into regulatory layers linked to pathogenicity, the functional consequences for host interaction and virulence phenotypes *in vivo* remain to be established. Finally, given that pathogenicity islands, such as the locus of LEE, are characterized by distinct GC content and codon usage (24), it will be important to determine whether variation in codon bias, both within LEE across clinical strains and across other horizontally acquired elements, interacts with tRNA modification dynamics to influence translational efficiency and virulence. Whether such mechanisms extend to other pathogenicity islands or mobile genetic elements in diverse pathogens remains an open question.
